# A lower sleep regularity index (SRI) is associated with relapse in individuals with alcohol use disorder following inpatient treatment

**DOI:** 10.1038/s41598-022-26019-y

**Published:** 2022-12-14

**Authors:** Jennifer J. Barb, Alyssa T. Brooks, Narjis Kazmi, Li Yang, Subhajit Chakravorty, Gwenyth R. Wallen

**Affiliations:** 1 Present Address: National Institutes of Health Clinical Center, Translational Biobehavioral Health Disparities Branch, 9000 Rockville Pike, Bethesda, MD 20892 USA; 2 Perelman School of Medicine-Cpl. Michael J. Crescenz VA Medical Center, Philadelphia, PA 19104 USA

**Keywords:** Risk factors, Human behaviour, Addiction, Translational research

## Abstract

The sleep regularity index (SRI) is used to measure an individual’s sleep/wake consistency over time. The SRI has been associated with certain health risks; to date, research investigating the relationship between the SRI and relapse in individuals with alcohol use disorder (AUD) is lacking. The aim of this work was to evaluate the SRI and relapse in individuals with AUD following inpatient treatment. Individuals with AUD (n = 77, mean age = 49.5 ± 10.86) were assessed for 28-days following discharge from an inpatient treatment program. Logistic regression was applied to examine the impact of SRI on relapse as the outcome variable of interest. Sleep quality was lower in individuals who relapsed compared to those who did not. Moreover, SRI scores were significantly worse in those who relapsed compared to those who did not. Over the entire patient cohort, lower weekly SRI scores were significantly correlated with longer weekly nap duration. Logistic regression model results indicated that the overall SRI was a significant predictor of relapse. The SRI represents a relevant aspect of sleep health and should be considered when assessing an individual’s sleeping patterns. Behavior based interventions related to the importance of individualized consistency in sleep and wake patterns may be particularly important for treatment seeking individuals with AUD not only during inpatient treatment, but also once these individuals have transitioned into their outpatient phase of recovery. These findings support the notion of SRI as a separate facet of sleep health worth investigating in at-risk, disease specific groups.

## Introduction

Alcohol use disorder (AUD) is a chronic disease characterized by the inability to control one’s drinking. AUD affects approximately 14.1 million people in the United States which is approximately 5.6% percent of the population^1^. AUD frequently co-occurs with other psychiatric disorders^2^ and sleep complaints and disorders are prevalent among individuals with problematic alcohol consumption^3^. A 2019 review by Koob & Colrain reported that “…*sleep disturbances, alterations of sleep architecture, and the development of insomnia are ubiquitous in AUD*”^4^ highlighting the impact of alcohol on sleep architecture^5^. In another review by Stein et. al. of 107 relevant articles studying associations between disturbed sleep and alcohol use, the authors highlighted the need for clinicians to evaluate alcohol use in individuals reporting sleep problems and most notably suggested that thorough treatment studies are needed in individuals with chronic alcohol use and insomnia^6^.

Although there is mixed evidence on whether sleep disturbance predicts or plays a major factor in relapse among individuals with AUD^3^, some evidence suggests that the use of alcohol as a hypnotic is associated with subsequent relapse following inpatient treatment^7^. In a 12 month observational study by Kolla et al., sleep disturbances (measured by PSQI) were not associated with relapse at 12 months post treatment however, as previousy mentioned, they did suggest an association with relapse when alcohol is used as a hypnotic. Alternatively, in a pre-clinical study in rats published in 2021 exploring the relationship between impaired sleep and alcohol relapse showed that decreased slow-wave sleep and sleep restriction during abstinence from alcohol may place individuals at risk for relapse but this work was conducted in a pre-clinical model and needs to be explored further in individuals with AUD^8^. Furthermore, in a larger prospective study of 80 treatment seeking individuals for AUD, the authors found that individuals with baseline depression, anxiety and sleep difficulties did not predict subsequent alcohol use^9^. Considering that these studies have various mixed findings, the idea that insufficient sleep can impact relapse needs further exploration. One large epidemiological study conducted on the general population in Australia reported that greater variability in sleeping behaviors such as irregular sleep/wake patterns and more frequent insufficient sleep quality and duration were associated with higher alcohol consumption and other negative lifestyle behaviors such as lower diet quality and more time sitting^10^. These negative lifestyle behaviors could have implications for individuals with AUD who have achieved sobriety during inpatient treatment and subsequently must re-integrate into their homes and communities without the structure of an inpatient treatment environment.

In addition to typical measures of sleep duration and/or quality, Phillips et al. developed the Sleep Regularity Index (SRI) in 2017, a measure which assesses daily variability of sleep and wake rhythms over a period of time^11^. Phillips and colleagues demonstrated that the SRI was associated with poorer subjective sleep quality and circadian misalignment among undergraduate students, a population that is known to have irregular sleeping habits. They reported that the SRI is not associated with daily sleep duration suggesting that the SRI measure is a different component of sleep health. This group examined the effect of light exposure on sleep regularity and found individuals with irregular sleep had significantly less daytime light exposure. Later, Lunsford-Avery et al.^12^ demonstrated the utility of the SRI in a sample of 1978 older adults and reported that the SRI was associated with increased cardiometabolic risk. Furthermore, this study demonstrated an association between greater sleep regularity measured by higher SRI values and increased stress and depression along with other sleep or circadian-related outcomes^12^. A preliminary study conducted by our group evaluated the association between SRI and demographic, clinical, and sleep-related outcomes in treatment-seeking individuals with AUD during inpatient treatment^13^. Our results showed that the SRI improved across four weeks of inpatient treatment among specific sub-groups of individuals while they abstained from alcohol use^13^. Furthermore, the SRI was associated with mood disorders, napping, and self-reported physical/mental exhaustion. Since the introduction of the SRI, there has been a growing interest in investigating the relationship between this index and other health outcomes such as alcohol consumption, reduced default mode network connectivity in the brain among healthy and young adults^14^, circadian and caloric intake in patients with Type 2 Diabetes^15^, and circadian and sleep regularity in older adults with Alzheimer’s Disease^16^, and individuals with current or remitted depression^17^.

Despite a large body of evidence supporting the bidirectional relationship between sleep disturbance and drinking among individuals with AUD and napping being a component of sleep disturbance in recovery^18^, to date there is no research examining the relationship between sleep regularity and relapse to drinking following discharge from inpatient alcohol treatment. Among those who seek inpatient treatment for AUD, the transition from a structured inpatient environment back to their homes and communities can be a stressful period^19^, that may disrupt their prior sleep schedule and regularity in inpatient setting and make their sleep disturbance worse.

Understanding predictors of relapse to drinking following abstinence is of interest to individuals with AUD, scientists, and clinicians. We hypothesize that individuals who relapse, especially upon discharge from a structured and supportive environment from an inpatient treatment program, may experience interrupted sleep patterns at a higher rate than those who do not upon completion of treatment for alcohol use disorder and this current work is aimed to explore this concept. This analysis builds on previous literature by assessing the relationship between SRI and relapse to drinking among individuals with AUD recently discharged from an inpatient treatment program. The primary aim of this work is to understand the relationship between relapse and the SRI on individuals who completed a 28-day inpatient treatment program for Alcohol Use Disorder.

## Methods

### Sample and procedure

This study was approved by International Review Board of the National Institutes of Health (NCT#02231840). All methods were performed in accordance with the relevant guidelines and regulations. All participants in the enrolled protocol provided informed written consent. Participants who met the criteria for AUD according to the Structured Clinical Interview for the Diagnostic and Statistical Manual of Mental Disorders (DSM-IV or DSM-5) (SCID) were first enrolled on a screening and assessment clinical trial (NCT#02231840) and admitted to the NIH Clinical Center for a 28-day inpatient AUD treatment program. Participants were eligible for the current clinical study “*Sleep Disturbance and Relapse in Individuals With Alcohol Dependence: An Exploratory Mixed Methods Study*” (NCT#02181569) if they were an inpatient for 21 days or more preceding discharge, not enrolled onto a pharmacologic intervention study, able to understand the study and provide informed consent, and willing to complete a follow-up visit in person or by phone/mail within four to six weeks of discharge from inpatient treatment. The current analysis focusing on the sleep regularity index following discharge from an inpatient treatment program represents a sub-analysis of the total sample and includes only participants who met inclusion criteria. The detailed methods and study population description have been previously published^19,20^. As part of the health and physical data collection, other substances used by the participants was collected and recorded.

### Measures

#### Clinical and alcohol related measures collected

Clinical and alcohol related measures were collected to characterize this population with alcohol use disorder. As part of the data collection process for the outpatient population, participants were instructed to complete diaries related to daily sleep habits and alcohol consumption post-discharge for 28 days. Daily sleep and symptom diaries were used to cross-validate semi-objective sleep data collected via actigraphy and to assess any alcohol intake during post discharge period. Patients were instructed to complete diaries once daily in the morning upon waking. We assessed relapse from patient report in the diary as any alcohol consumed during that time. Upon diary inspection, individuals were dichotomized in to either the relapse group or non-relapse group based on whether they consumed any alcohol during the 28-day outpatient follow up. Participants were instructed to add up the number of alcoholic drinks consumed per day, which was used to calculate the outcome variables used for this analysis; Total number of drinking days (any alcohol), average drinks per day and number of heavy drinking days (4 or more drinks for females, five or more drinks for males). These variables were calculated only for the subset that reported any alcohol consumption during the post discharge study time period. Additionally, during the post-discharge data collection time period, patient reported measures of alcohol cravings using the *Penn Alcohol Craving Scale (PACS)*^21^, and anxiety and depression using two subscales (Brief Scale for Anxiety; BSA & Montgomery Asberg Depression Rating Scale; MADRS) of the *Comprehensive Pathological Rating Scale (CPRS)*^22–24^ were collected*.* As part of the data collection process, these variables were collected if a participant returned to the Clinical Center for follow-up visits. Since not all participants returned to the Clinical Center, the CPRS anxiety and depression measures are not complete for all participants included in this analysis. In addition to the patient reported subjective measures, we also used a SCID diagnosis of mood and anxiety disorders administered by trained professionals on the inpatient unit in the NIH Clinical Center^25^. A more detailed description of the study clinical measures and their timing of administration are provided in Supplemental Table 1.

**Table 1:** Patient population demographics (N^a^ = 77).

	Did not relapse (n = 59)	Relapse (n = 18)	p-value
**Characteristic**	Mean (SD)		
Age	51.1 (10.1)	44.4 (12.1)	**0.021**
**Sex**	Number (%)		
Male	42 (71.2)	11 (61.1)	0.419
Female	17 (28.8)	7 (38.9)	
**Race**			0.182
White	35 (59.3)	11 (61.1)	
Black or African American	19 (32.2)	3 (16.7)	
Other^b^	5 (8.5)	4 (22.2)	
**Ethnicity**			0.377
Non-Hispanic	56 (94.9)	16 (88.9)	
Hispanic	2 (3.4)	2 (11.1)	
Unknown	1 (1.7)	0	
**Marital status (n = 76)**			0.053
Married	15 (26.3)	1 (5.6)	
Other^c^	42 (73.7)	17 (94.4)	
**Anxiety disorders (SCID IV/V)**^**d**^			0.300
Yes	14 (23.7)	6 (33.3)	
**Mood disorders (SCID IV/V)**^**d**^			0.353
Yes	15 (25.4)	6 (33.3)	
**Polysubstance use**			
Yes	17	9	0.096
**Alcohol consumption**	Mean (SD) # of drinks reported		
Week 1 (n = 18)	0	4.1 (6.7)	
Week 2 (n = 18)	0	12.6 (19.1)	
Week 3 (n = 17)	0	21.7 (32.1)	
Week 4 (n = 15)	0	29.1 (46.4)	
Total (28 days)	0	61.4 (96.7)	

Significant values are in [bold].

^a^Total number of participants with valid data and included in the analysis based on filtering rules.

^b^Other includes American Indian, Asian, Multiracial and other.

^c^Other includes divorced, separated, single, or widowed.

^d^Diagnosis of one or more disorder according to Structured Clinical Interview for DSM-IV/5 Disorders.

#### Subjective sleep measures collected

Patient-reported measures of subjective sleep quality using the *Pittsburgh Sleep Quality Index (PSQI)*^26–28^, daytime functioning using *Epworth Sleepiness Scale (ESS)*^29,30^, sleep related cognitions including faulty beliefs using *Dysfunctional Beliefs and Attitudes about Sleep (brief version; DBAS-16)*^31^, and individual level of confidence in performing sleep initiating behaviors using *Self-Efficacy for Sleep (SE-S)*^32,33^ were assessed. For further details on these subjective measures, please see Supplemental Table 1. The subjective habitual sleep efficiency used in this analysis is comprised from one of the 7 subscales of the PSQI and is calculated as a percentage of patient reported number of hours slept divided by the total number of hours reported spent in bed. All self-reported sleep measures were recorded within 7 days of discharge and within 4–6 weeks post-discharge.

#### Objective sleep measures collected

##### Respironics Actiwatch Spectrum plus actigraphy data measures collection methodology

Actiwatches are small data loggers that are worn on the non-dominant wrist and record digitally integrated measures of gross motor activity. These devices contain accelerometers and light sensors to objectively assess sleep and physical activity of the individual wearing them. Prior studies have demonstrated actigraphy’s high sensitivity with moderate accuracy for assessing sleep parameters in populations with normal and disturbed sleep when compared to polysomnography^34–36^. Our participants were instructed to begin wearing the Actiwatch Spectrum *Plus* (Philips Respironics) continuously from the time of enrollment into the sleep study (beginning within seven days prior to their planned discharge date) until the fourth week after discharge, which typically coincided with the final visit for follow-up assessments.

After downloading the Actiwatch data from each participants’ watch (using Philips Actiware 6.0.9 software, https://www.usa.philips.com/healthcare/sites/actigraphy/solutions/actiware), the study team screened the files for any malfunctioning watches, corrupt data or required adjustments using daily self-reported sleep diaries for cross-validation. Major rest intervals were marked using programmed functions of the software and were adjusted (inserted a new sleep interval OR extended, shortened, or split an existing one) only if any discrepancy was noticed based on sleep diary data. A total of 22 of the 77 participants did not require any modifications in sleep intervals marked by Actiware. adjustments of up to 9 days of actigraphy recorded sleep intervals were made by the group as part of the cleaning process for 53 participants. There were 5 participants who required modifications in sleep intervals for 10 or more days with no more than a maximum of 23 days. Minor rest intervals (naps) were manually entered based on activity patterns during the day only if the participant mentioned taking a nap on that day in their sleep diary. The recording of a nap was not based on a time of day but was based on a short interval of rest independent of the major rest interval (typically nighttime sleep). For all instances where the two group members did not agree with a marked interval, the questions were brought to the whole team for a consensus meeting. Weekly averages of the following variables were calculated from cleaned actigraphy data: sleep duration, wake after sleep onset time (WASO), sleep onset latency (SOL), sleep efficiency, and nap duration for each 24-h period. Actigraphy based sleep efficiency is calculated as the percentage of sleep during a given rest interval which is the time in bed (i.e., total sleep time divided by interval duration minus total invalid time (sleep/wake)).

##### Data cleaning and Sleep Regularity Index calculation

The SRI python script (https://github.com/mengelhard/sri) was adapted into the JMP (version 14, SAS Headquarters, Cary, NC, www.jmp.com) Scripting Language (JSL) with additional customization considerations specific to this project. Much of the workflow was followed as described in Brooks et al.^13^ with a few other edits described herein. Actigraphy data were recorded starting at 5:00:00 PM on the day of discharge and collected for the next 28 days (24-h intervals with 1440 epochs for each day where an epoch is 1 min). One matrix of interval status columns and epochs for each participant was saved and used to calculate the weekly SRI and total SRI scores (across 28 days, if available). For this analysis, each participant was required to have at least seven days of actigraphy data. The customized JSL script calculates a total average SRI over 28 days (when available) and a seven-day average SRI for weeks 1,2, 3 and 4 (i.e., days 1–7, days 8–14, days 9–21 and days 22–28, respectively). The formula for the SRI calculated was first introduced by Lundsford-Avery et.al and was used in a previous publication from our group^12,13^. The formula to calculate the SRI based off of the publication from Lundsford-Avery et. al^12^ is shown below:

$$SRI=-100+\frac{200}{M(N-1)}{\sum }_{j=1}^{M} \sum_{i=1}^{N-1}\delta ({s}_{i,j},{s}_{i+1,j})$$

where $\delta ({s}_{i,j},{s}_{i}+ {1}_{,j})$ =1 if ${s}_{i,j}$=${s}_{i+1,j}$ and 0 otherwise.

The SRI can be interpreted as higher values indicate more regular sleep/wake patterns and lower values indicate irregular sleep/wake patterns. For example, if a person goes to sleep and wakes up at the same time every day during an assessment period, this individual will have a SRI close to (or exactly) 100. The total SRI and weekly SRI was calculated by averaging all SRIs for the specific time interval (i.e., total for 28 days denoted total SRI and weekly for week 1, week 2, or week 3 and week 4). To assess the completeness of the actigraphy data, the script will calculate the total number of ‘EXCLUDED’ minutes recorded by the watch for each day.

##### Data filtering

An individual was included in the analysis if they had at least seven days of actigraphy data captured. In addition to this requirement for inclusion, there could be no more than 1 day with 6 or more excluded hours over the 28-day assessment period. The justification behind this was based on the allowance of removing the watch for activities such as showering, bathing, and/or swimming. This filter removed 43 participants, leaving a subset of 77 participants to be included in this analysis.

##### Light exposure data

Daily light exposure was investigated in this patient cohort to determine if an association could be observed between the SRI and daytime and nighttime light exposure as was previously reported in Philips, et al^11^. Daily white light exposure was collected for each patient per 24-h period (comprised of 1440-min epochs). Light exposure data capture for each patient began at 10:00 PM on the day of discharge. A binary variable of 0 and 1 indicating no light exposure or light exposure for each epoch was created. If the watch detected ≥ 250 lm of white light, the variable was coded as 1 and if the watch detected < 250 lm of white light, the variable was coded as 0. Three white light exposure variables were created as follows by summing over the binary (0,1) variable for three different time intervals: total 24-h white light exposure, total 12 h of white light exposure during the interval of 10:00 AM to 9:59 PM (for clock day) and a total 12 h of white light exposure during the interval of 10:00 PM to 9:59 AM (for clock night). Clock day and clock night time intervals were chosen based on previous literature^11,12^. The result of this calculation gives a total number of minutes of white light exposure for each time interval of interest. The three light exposure variables were averaged over the entire 28 days (total) or were averaged weekly.

### Statistical analysis

The race and marital status demographic variables were recoded into the following categories: race was categorized as ‘White’, ‘Black/African American,’ and ‘Other’ (American Indian, Asian, Multiracial and other); marital status was re-coded into ‘Married’ and ‘Other’ (divorced, separated, single, or widowed). Relationships between relapse and total SRI, and demographic variables (age, gender, marital status, and race/ethnicity), alcohol craving (PACS), sleep efficiency, self-reported sleep quality (PSQI), excessive daytime sleepiness (ESS), weekly nap duration, and actigraphy-recorded sleep and physical activity variables were examined with Chi-squares, Student’s t-tests, Pearson correlation analysis, one-way ANOVA, and Wilcoxon/Kruskal–Wallis tests. Variables significantly associated with relapse were included as covariates in the stepwise logistic regression model that evaluated the effect of overall SRI on relapse. The first step included total SRI only. Demographic variables that were significantly different between relapse and non-relapse were added in the second block and sleep related variables were added in the third block.

Weekly SRI scores were investigated over the entire patient cohort to assess a linear change in the SRI over the four weeks. Linear mixed models were used to evaluate whether the weekly SRI scores changed between weekly drinking status groups (relapsed vs. no relapse) over time and whether the weekly SRI scores changed between the overall relapse status groups over time. Different residual error covariance matrices were compared and the one with smallest Akaike’s Information Criterion (AIC) and Schwarz’s Bayesian Criterion (BIC) was chosen as the final model. Linear mixed models assessing the impact of napping on the SRI between the two groups was assessed with the following fixed effects: relapse, week, nap duration, relapse by week interaction and nap duration by relapse interaction. Data analyses were performed using JMP Data Discovery Statistical Software (SAS Institute Inc., Cary, NC, USA), the IBM SPSS Statistics software (Armonk, NY, USA), and SAS 9.4 (SAS Institute Inc., Cary, NC, USA). A p-value of < 0.05 was considered statistically significant.

## Results

### Study population demographics

This study sample (n = 77) was mostly male (67.9%), White (60.3%), and non-Hispanic (93.6%) with a mean age of 49.41 ± 10.84 (Table 1). Twenty percent of the individuals in this sample were married, whereas 80% of the individuals were either divorced, separated, single, or widowed, and 27% had either a mood and/or anxiety disorder based on the SCID-IV/V. Individuals who relapsed were significantly younger 44.4 ± 12.08 than those who did not relapse 50.9 ± 10.07 (*p* = 0.02). There were no significant differences between individuals who relapsed and individuals who did not relapse in sex, race, ethnicity, marital status or SCID-IV/5 diagnosed mood/anxiety disorders. A total of 26 participants reported using substances (polysubstance use) other than alcohol including marijuana, cocaine, crystal methamphetamine, heroin, crack cocaine and prescription drugs upon entry into the treatment program. The most frequently used substance among the 26 individuals was marijuana (n = 20). Furthermore, 9 of the 18 individuals who reported a relapse event reported using other substances.

### Objective and subjective sleep measures

**Figure 1:**
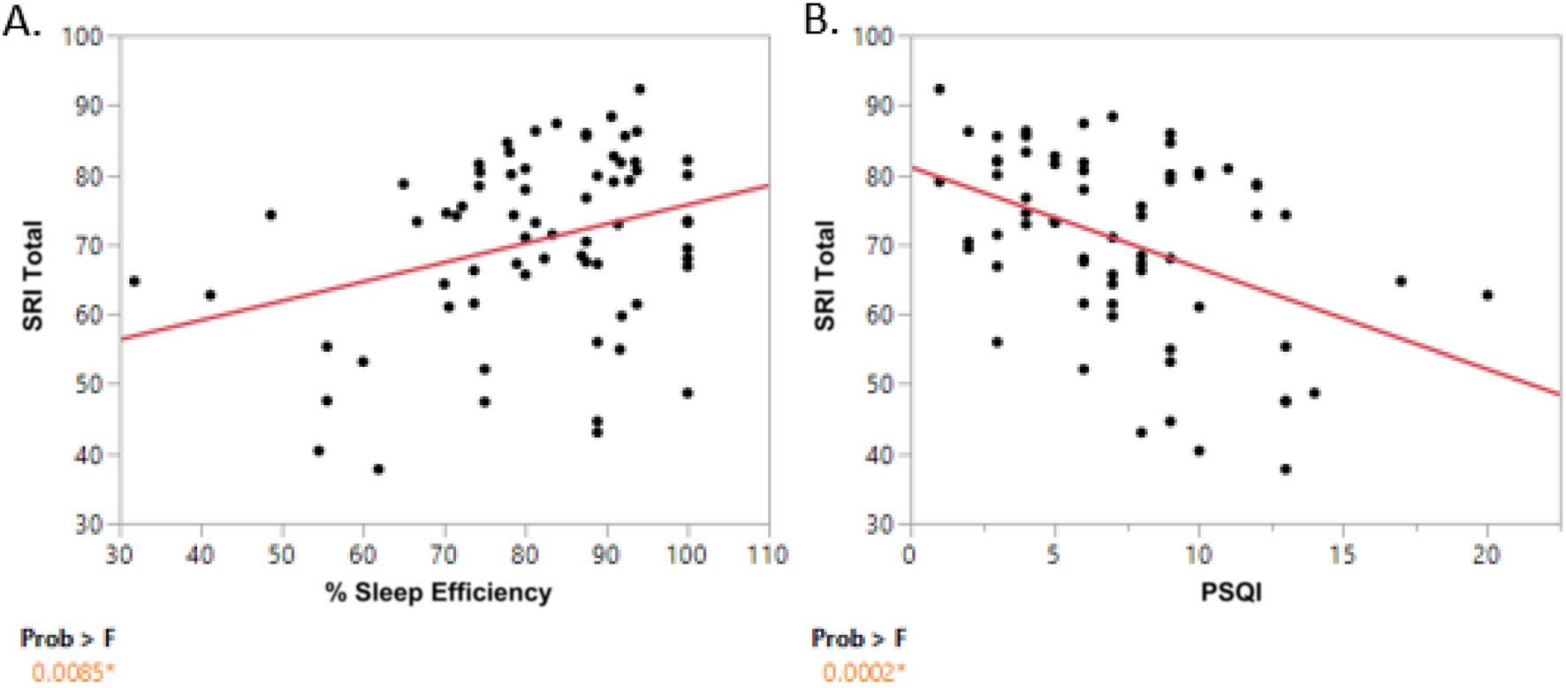
Bivariate plot of total SRI versus percent sleep efficiency and sleep quality. Total SRI (y-axis) versus (**A**) patient reported percent sleep efficiency (x-axis). A significant positive correlation between total SRI and sleep efficiency (p = 0.008) is observed and (**B**) sleep quality measured by PSQI (x-axis). A significant negative correlation between total SRI and sleep quality (p < 0.001) is observed.

The subjective sleep measure averages over the entire patient cohort were as follows: average total SRI score was 70.29 ± 12.57, average PSQI (administered 4–6 weeks post-discharge which assesses at least the last 2 weeks of sleep) was 7.29 ± 3.82 and average subjective sleep efficiency (percentage) was 81.19 ± 14.58. A PSQI value greater than 5 is indicative of poor sleep. Actigraphy sleep measures, calculated from the Actiware software are presented as weekly averages in Table 2. Over the entire cohort, the total SRI score was significantly positively correlated with the subjective % sleep efficiency (r = 0.31, *p* = 0.008, Fig. 1A), and negatively correlated with PSQI (r = − 0.43, *p* < 0.001, Fig. 1B), indicating that higher total SRI scores were associated with lower sleep disturbance, as measured by the PSQI. There were no significant relationships observed between total SRI and the other subjective measures of sleep (i.e., hours in bed, DBAS, EPS and SES; Supplemental Fig. 1).

**Table 2:** Actigraphy based sleep and light exposure variables.

Actigraphy measures	Week 1 (n = 77)	Week2 (n = 74)	Week 3 (n = 73)	Week 4 (n = 72)	Over 28 days (n = 77)
SRI	70.23 ± 18.40	70.37 ± 14.08	70.48 ± 13.84	71.81 ± 13.40	70.29 ± 12.57
Nap duration (min)	126.99 ± 103.09	117.21 ± 73.19	103.03 ± 45.57	107.41 ± 84.62	
Sleep efficiency (%)	81.24 ± 6.54	80.76 ± 7.41	80.69 ± 8.05	80.84 ± 8.88	
WASO (min)	61.01 ± 21.20	64.09 ± 25.34	62.73 ± 24.56	59.68 ± 23.49	
Sleep duration (min)	453.98 ± 75.22	463.76 ± 81.20	459.40 ± 89.88	451.28 ± 79.19	
Sleep onset latency (min)	15.57 ± 16.31	15.80 ± 10.73	15.79 ± 14.17	18.20 ± 15.72	
**Light exposure (min)**					
Total 24-h	89.4 ± 76.2	83.25 ± 83.61	86.31 ± 78.64	89.85 ± 76.71	86.31 ± 71.4
Total daytime	75.69 ± 64.28	72.09 ± 71.42	73.46 ± 65	77.27 ± 64.38	73.96 ± 59.65
Total nighttime	13.98 ± 14.83	11.24 ± 14.8	12.85 ± 16.47	12.58 ± 16.72	12.43 ± 13.57

Data presented as Mean ± Standard Deviation. Light exposure is number of epochs (minutes) with ≥ 250 lumen over time interval. Daytime corresponds to the interval 10AM to 9:59PM; nighttime corresponds to the interval 10 PM to 9:59 AM.

### Daily day and night light exposure assessment

Average light exposure (≥ 250 lm) for 24-h was assessed to investigate if there was an effect on weekly and/or total SRI scores (Table 2) since a previous publication showed an association with irregular sleep and lower daytime light exposure^12^. There were no associations observed between total SRI and average light exposure over a 24-h interval (r = 0.08,* p* = 0.471) nor at weekly intervals. Average nighttime light exposure (r = 0.15,* p* = 0.174; Supplemental Fig. 2A) and daytime light exposure (r = 0.06, *p* = 0.588; Supplemental Fig. 2B) were not associated with total SRI score.

### Alcohol consumption relapse events

Eighteen individuals reported consuming any amount of alcohol over the 28-day post-discharge period (Supplemental Fig. 3A). The total number of drinks consumed, based on patient diaries, ranged from 1 to 342 over the 28-day assessment period (Supplemental Fig. 3B). Among the individuals who relapsed, the number of days until drinking occurred following discharge ranged from the first day to the 18th day post-discharge (Supplemental Fig. 3C). Of the individuals who relapsed, 9/18 consumed any alcohol within the first week post-discharge, 5/18 individuals consumed any alcohol within two weeks post-discharge and the remaining 4/18 individuals consumed any alcohol by the third week following discharge. Among those who relapsed, the average number of heavy drinking days was 5.39 ± 7.86, and the weekly drinks consumed increased over the four weeks of the assessment period with 4.1 ± 6.7 drinks during the first week to 29.1 ± 46.4 drinks during the fourth week (Supplemental Fig. 3D). While relapse was defined as any alcohol consumed over the 28-day assessment period, it is worth noting that the average percent of days abstinent among the relapsed group ranged from 3 to 96% of the time (67% ± 30%). Additionally, when considering participants who reported using other substances in addition to alcohol upon entry into the inpatient treatment program, 9 of the 18 individuals who relapsed used other substances while 17 of the 59 who did not relapse used other substances. This difference was not significant since the proportion of individuals who reported using other substances did not differ by relapse status (*X*^2^ (1, *N* = 77) = 2.67, *p* = 0.096). Relapse status was investigated across the PACS at week 1, week 2 and week 4 to determine if cravings might have a relationship to whether an individual in this cohort consumed alcohol or not. There was no difference observed between relapse and non-relapse in PACS at week 1 (*p* = 0.895), week 2 (*p* = 0.077) or week 4 (*p* = 0.918). Furthermore, no relationship was observed with other alcohol related variables such as days until a relapse event occurred and percent of days abstinent.

### SRI and average nap and sleep duration

**Figure 2:**
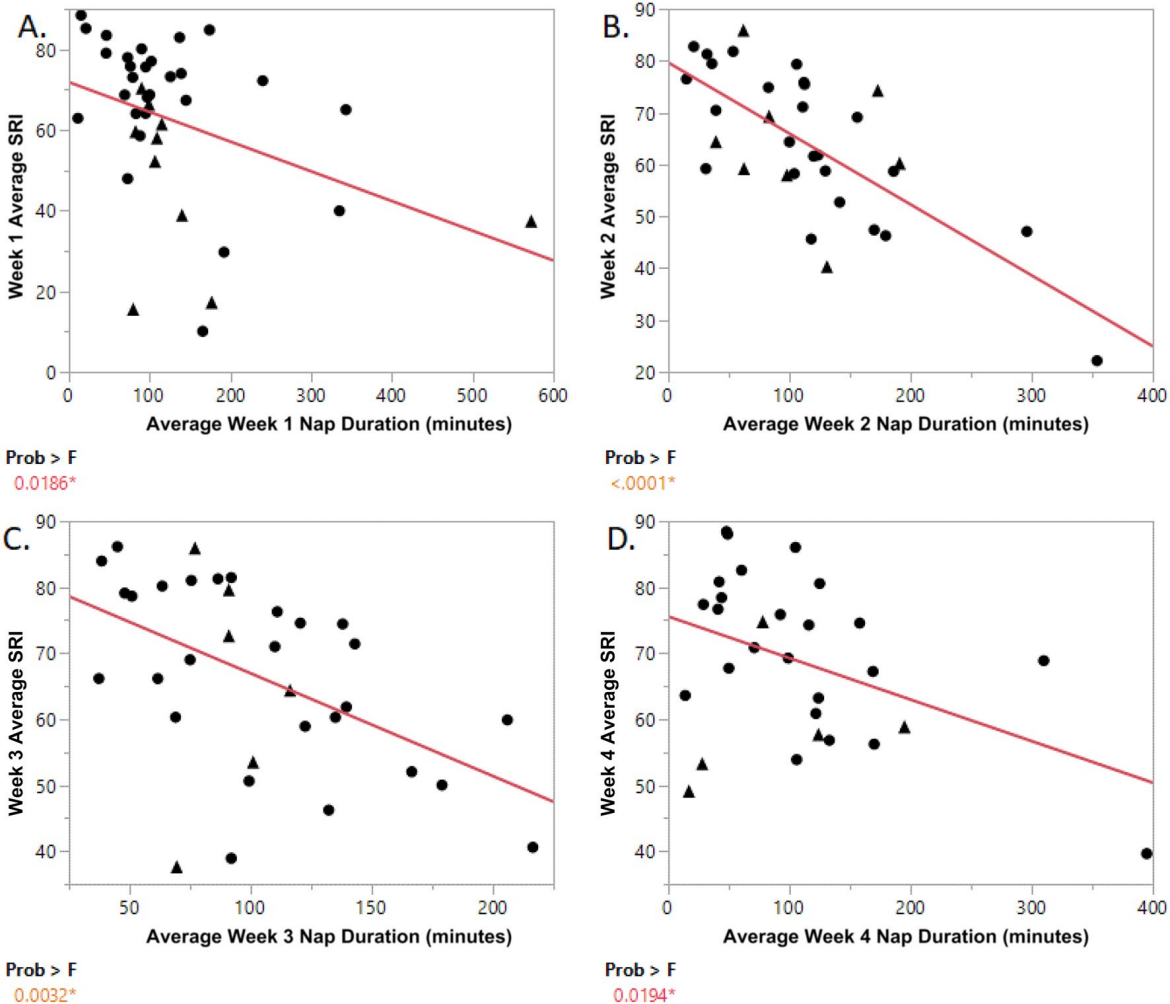
Weekly SRI versus weekly nap duration. Weekly SRI versus weekly average nap duration. (**A**) Week 1 SRI (x-axis) versus average week 1 nap duration (y-axis). Pearson correlation r = − 0.38, *p* = 0.018. (**B**) Week 2 SRI (x-axis) versus average week 2 nap duration (y-axis). Pearson correlation r = − 0.71, *p* < 0.0001. (**C**) Week 3 SRI (x-axis) versus average week 3 nap duration (y-axis). Pearson correlation r = − 0.49, *p* = 0.003. (**D**) Week 4 SRI (x-axis) versus average week 4 nap duration (y-axis). Pearson correlation r = − 0.43, *p* = 0.019.

Daily napping was averaged over weekly intervals in order to investigate whether the SRI and nap durations were associated, as previously reported^13^. Over the entire cohort, each weekly SRI score was significantly negatively associated with the respective weekly average nap duration (r = − 0.38, *p* = 0.018; Fig. 2A); (r = -0.71, *p* < 0.001; Fig. 2B), (r = − 0.49, *p* = 0.003; Fig. 2C), and (r = − 0.43, *p* = 0.019; Fig. 2D), week 1 through week 4 respectvely. Additionally, total daily sleep duration was calculated for each individual and was averaged over weekly intervals and investigated against the total and weekly SRI. There were no significant associations observed between total SRI and total sleep duration over 28-days nor were any significant associations observed between weekly SRI scores and weekly sleep duration.

### SRI, subjective sleep measures and relapse

Subjective percent sleep efficiency was significantly lower in individuals who relapsed (74.32 ± 17.24) compared to those who did not (83.11 ± 13.32) (*p* = 0.036) (Supplemental Table 2). Other subjective sleep measures did not significantly differ between relapse and non-relapse (Supplemental Table 2).

**Figure 3:**
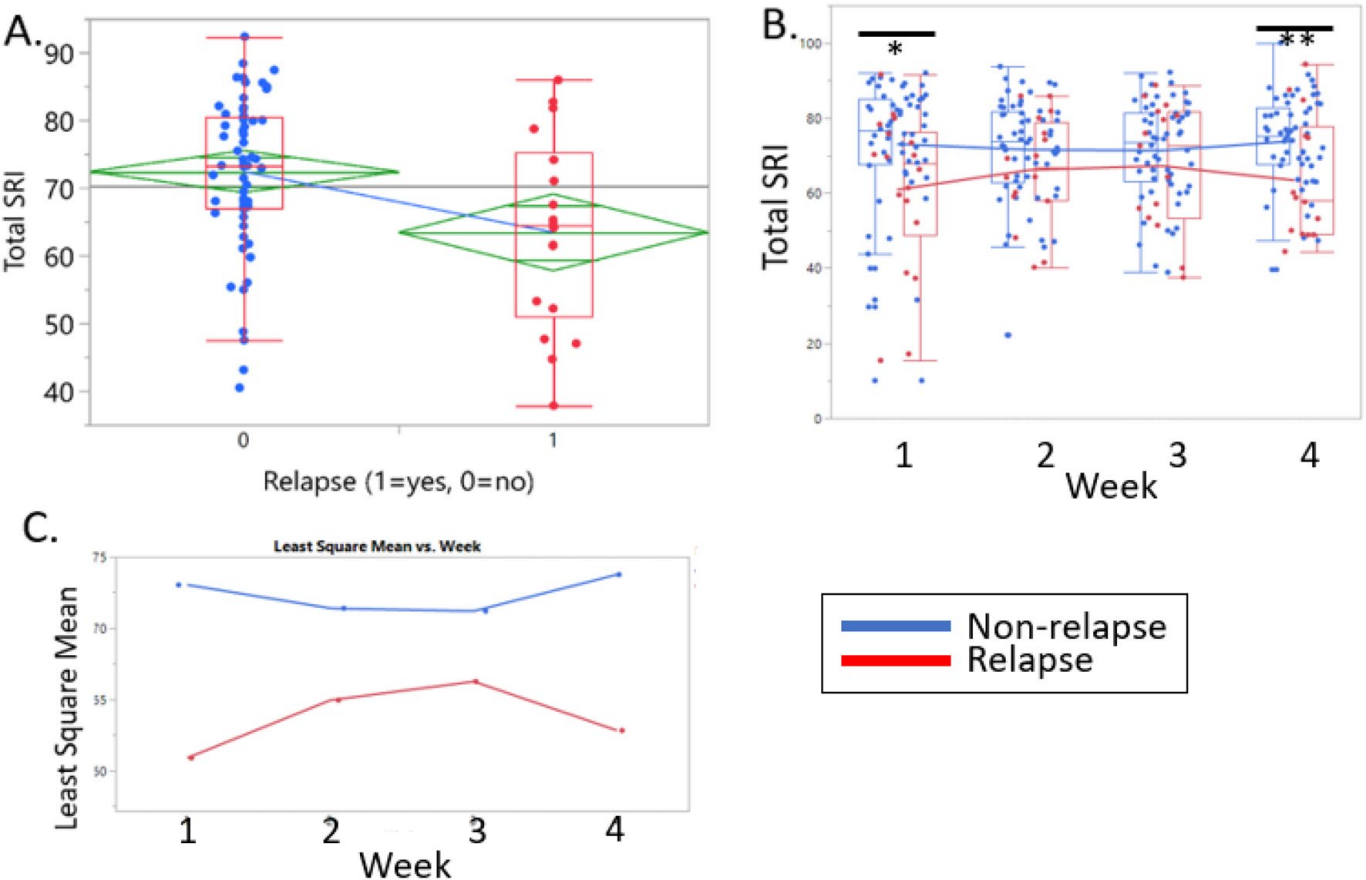
Total SRI, weekly SRI, and least square mean between relapse group and no relapse group. Total and Weekly SRI in relapsers (red) and non-relapsers (blue). (**A**). One-way plot showing total SRI (y-axis) in non-relapsers ‘0’ (N = 60, µ = 72.36, blue dots) and relapsers ‘1’ (N = 18, µ = 63.38, red dots) (x-axis). The line near the center of each green diamond in Fig. 1A represents the group mean for the specified group on the x-axis. Significant difference in total SRI between relapsers and non-relapsers (Independent samples t-test *p* = *0.007*). (**B**). Overlay Box Plots of weekly SRI values (week 1, week 2, week 3, and week 4) in non-relapsers (blue dots) and relapsers (red dots). Red line indicates the weekly SRI average for relapsers and blue line indicates the weekly SRI averages of non-relapsers. Independent samples t-test at each week showed signficant difference at week 1 (*p* = *0.01*) and week 4 *(p* = *0.008*). (**C**). Least square means over each week between relapsed and non-relapsed.

Linear mixed models assessing the change of SRI over the four weeks showed no significant difference over the entire cohort (*F*_3, 76_ = 0.32, *p* = 0.81) nor significant interactions between relapse status and time (*F*_3, 76_ = 2.66, *p* = 0.054). The group who relapsed had significantly lower SRI scores (63.38 ± 14.16, n = 18) than those who did not relapse (72.35 ± 11.48, n = 59), (F_1, 76_ = 6.52, *p* = 0.013) (Fig. 3A). More specifically, the group who did not relapse had higher SRI scores at week 1 (*p* = 0.0131) and at week 4 (*p* = 0.0042) compared to those who relapsed (Fig. 3B, C). No significant differences were found in SRI scores at week 2 (*p* = 0.1029) and week 3 (*p* = 0.2105) between the two groups. Total SRI score alone was a significant predictor of relapse (*p* = 0.041) (Table 3). When the stepwise multiple logistic regression model was run after controlling for age, the overall SRI was still a significant predictor of relapse (*p* = 0.031) (Table 3). When the subjective sleep efficiency was added to the model, the overall SRI was not a significant predictor of relapse (*p* = 0.11). Among those who reported any drinking, there were no significant association between total SRI and days until relapse (*r* = 0.04, *p* = 0.85).

**Table 3:** Stepwise Logistic regression models of SRI on relapse status.

Variable	OR	95% Confidence Interval for OR		p-value
		Lower	Upper	
Step 1				
Total SRI	0.954	0.913	0.998	**0.041**
Step 2				
Total SRI	0.948	0.904	0.995	**0.031**
Age	0.953	0.903	1.006	0.084
Step 3				
Total SRI	0.958	0.910	1.010	0.110
Age	0.949	0.897	1.004	0.071
Subjective Sleep efficiency	0.968	0.929	1.009	0.122

Significant values are in [bold].

**Figure 4:**
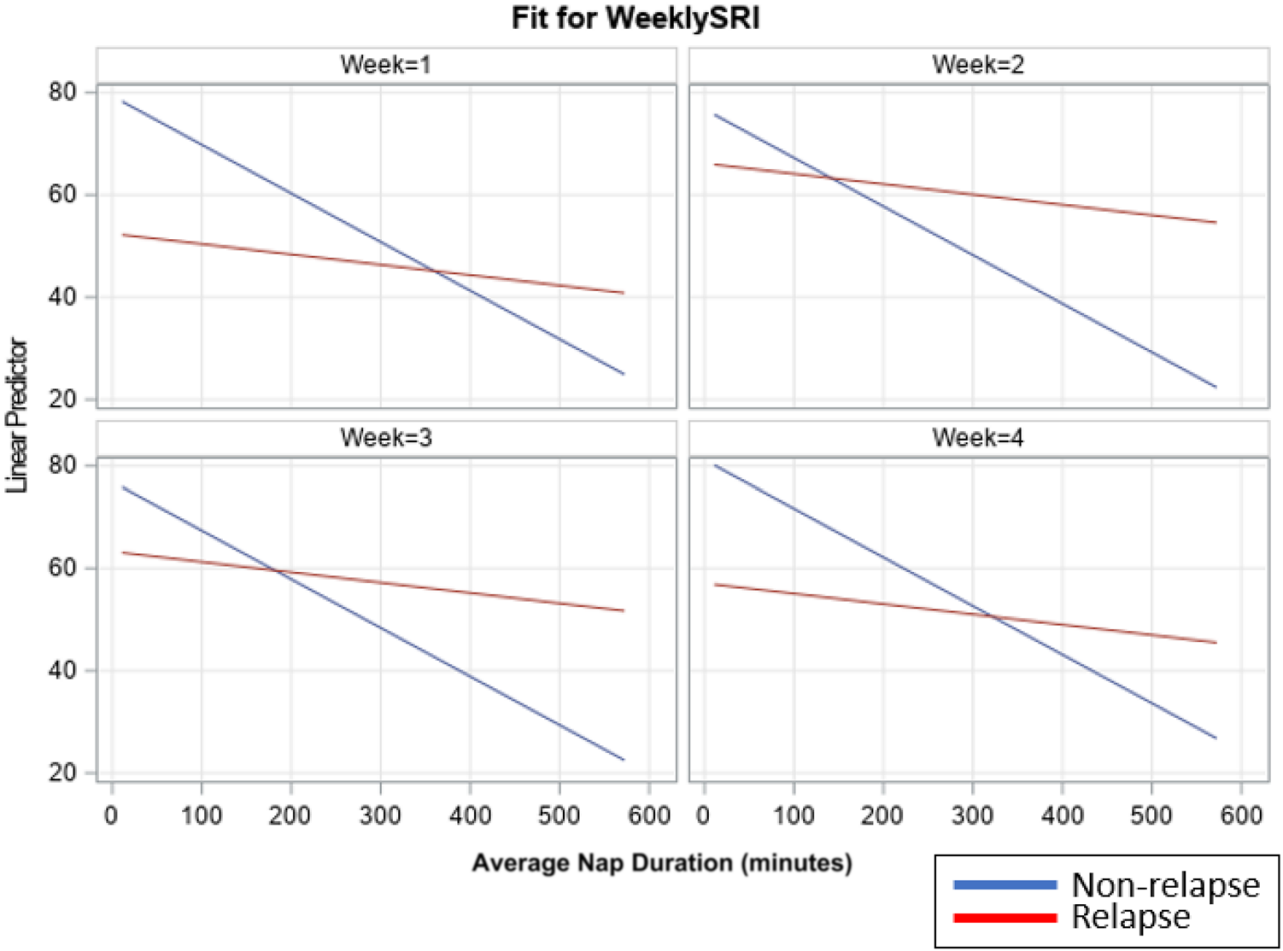
Weekly SRI versus Weekly Nap Duration between relapse and non-relapse. Weekly SRI and mean nap duration in individuals who relapsed (red) and those no did not (blue). The y-axis indicates the model predicted weekly SRI and the x-axis indicates the average nap duration over the group. The slope of the non-relapsed group is steeper indicating that napping had a higher impact on the SRI in individuals who did not relapse.

When the impact of napping on the SRI was investigated between the individuals who relapsed versus those who did not, the impact of nap duration on SRI was significantly different between the groups (*F*_1, 70_ = 5.79 *p* = 0.019, Fig. 4). In other words, the impact of napping on the SRI was higher among the individuals who did not relapse.

## Discussion

This analysis explored sleep and relapse events in individuals with AUD following a 28-day inpatient treatment program. Both subjective and actigraphy based objective sleep measures were included and explored within the patient cohort. The SRI investigating consistent sleep and wake cycles in relation to relapse was a focus of this work; however, other sleep related measures were included and reported. Specifically, the relationship between SRI and demographic, clinical, sleep-related variables and daily light exposure were investigated. The occurrence of relapse along with the daily number of drinks, days until a relapse event and total percent abstinence during the 28-day outpatient assessment period was reported and investigated. The rate of relapse in this cohort was 23% which is in line with what has been previously reported where individuals relapse at a rate between 20 and 50% following treatment^37^.

Our findings demonstrate a significantly lower total SRI score in individuals with AUD who experienced a relapse event compared to those who did not following an inpatient treatment program for AUD. Moreover, in the logistic regression model, SRI was a significant predictor of relapse when considered alone and when age was controlled for. Overall SRI was significantly associated with subjective sleep efficiency and sleep quality in this cohort of patients; however, overall SRI was not significantly associated with the objective sleep efficiency as recorded from the Actiwatches. When total nap duration was considered at weekly intervals over the four weeks, there were consistent negative correlations with weekly SRI scores, which is consistent with what we previously reported in our inpatient setting cohort^13^. Not surprisingly, longer nap durations were associated with lower SRI scores over the entire patient cohort. Interestingly, when the effect of napping on SRI was considered between relapse groups, the impact of naps on SRI was higher in individuals who did not relapse. We do not fully understand why napping affected the non-relapse group in this way, we could, however, hypothesize that it could be because that group had a larger sample size (n = 59) to test this association in and therefore the statistical rigor of the test was improved. Furthermore, we could speculate that napping might directly impact regular sleep patterns of individuals who do not use alcohol to cope with sleep disruption. While this finding was unexpected between the two groups, further follow-up on this topic is warranted.” The average total SRI for this outpatient sample of 77 individuals was 70.29, which was lower than that seen in our previous analysis (n = 124, 76.95), where we assessed the SRI in an inpatient setting^13^. The “structured” environment in the inpatient setting, with regularly scheduled meals and “lights out” times, may have influenced the higher average SRI in the inpatient sample in contrast to an unstructured environment after their discharge in the outpatient sample. This outpatient cohort had an average PSQI of 7.29, also different than what was reported in an inpatient sample (PSQI average: 6.46), indicating that these individuals had higher levels of sleep disturbance. Finally, the average objective and subjective percent sleep efficiencies in this cohort were 80.82% and 81.2%, respectively, which are slightly lower than a normal sleep efficiency in healthy individuals of 85%^38^.

The SRI represents a different aspect of how sleep, and specifically sleep regularity, might be an important sleep-related behavioral factor to consider when designing patient treatment programs during recovery. There was a significant inverse relationship between napping duration and SRI in both our inpatient analysis and the current outpatient analysis following treatment. Daily average sleep duration has always been an important component of sleep health in that it is recommended that adults get between 7 and 9 h of sleep per night^39^. The individuals in this study had about 7.63 ± 1.12 h of sleep which is in line with what is recommended. When total sleep duration was compared to the SRI, there was not a significant association over the entire patient cohort, confirming what was reported by Philips et. al in 2017^11^. This finding gives insight to the idea that sleep duration and a consistent sleep/wake schedule are not associated and that other measures of sleep are more important when considering healthy sleep habits.

Previous research has shown that light exposure can contribute to internal circadian sleep cycles^40–42^. Philips et al., showed that daily light exposure was significantly different in their group of irregular sleepers when compared to their group of regular sleepers; in fact, they noted that their irregular sleeping group was exposed to significantly less day-time light. We investigated total light exposure with respect to three-time intervals (24 h, 12 h during daytime, 12 h during nighttime) and found no significant association with light exposure on the SRI in any of the time intervals assessed (Fig. 3). Interestingly, the overall average 24-h daily light exposure was 86 min which equates to only one hour and 26 min of light. This low amount of total light exposure in this dataset could be due to the filtering criteria applied, or that a participant wearing a long-sleeve shirt could affect the amount of light detected by the watch contributing to some error artifacts from the device itself. In a future study, this could be verified by validating lumen exposure in a controlled setting and with or without a shirt covering the watch.

These findings, particularly the difference of the SRI between the relapse groups, may represent an important facet of sleep health with respect to a regular sleep and wake schedule that may help an individual maintain sobriety post-discharge from inpatient treatment for AUD. The SRI is a relatively new method of exploring the relationship between sleep quality and circadian misalignment and how this misalignment may contribute to relapse in individuals with AUD. While it makes sense conceptually that daytime napping might increase the variability of sleep timing, this provides further support for behavioral sleep interventions (including cognitive behavioral therapy for insomnia-CBT-I), which generally discourages daytime napping. A treatment plan that includes advice on achieving a more structured sleep schedule, such as a consistent sleep and wake time schedule may be particularly important for individuals with AUD who are discharged from inpatient programs or those preparing to transition out of such programs.

### Strengths and limitations

Several methodological strengths helped us gather particularly unique, nuanced data: as with our previous studies, we combined objective and subjective assessments of sleep, and using daily sleep and symptom diaries allowed us to capture daily drinking data for most participants in the absence of a Timeline Follow-Back. However, several possible limitations are worth acknowledging. It is important to note that differences in findings between inpatient and outpatient/residential AUD treatment may reflect a particularly stressful time of transition for many. These additional stressors may limit the interpretation of the associations between SRI and relapse and include, but are not limited to, going from a structured treatment milieu with individualized support, to having virtually no structure or support. Despite these difficult transitions, patients are often expected to follow their recovery goals and maintain abstinence while often returning to the environments in which they previously lived. These very struggles with transitions between inpatient and post-discharge environments have been described by individuals with AUD and previously published in a mixed methods study by our team^19^.

Much of the literature on sleep disturbance and alcohol use is focused on insomnia. We did not measure insomnia; rather, we assessed global sleep disturbance via the PSQI. Because of low variability in relapse status, we treated relapse as a dichotomous outcome, but it is possible that there is a dose–response relationship between alcohol consumption and sleep regularity. Furthermore, we also categorized individuals into the relapse category if they consumed any alcohol at all during the assessment period. This particular categorization into a relapse event could be controversial clinically as relapse events such as the amount of alcohol consumed, the average alcohol consumed over a period and the percent of time abstinent may be taken into account to decide whether a relapse event truly occurred by the individual. The anxiety and depression CPRS measures were collected during a post-treatment in person follow up appointment. Not all participants from whom actigraphy data was collected were able to return for inpatient follow-up appointments; therefore, the data for these measures are incomplete and were not assessed in the present analysis. Finally, our results cannot be generalized to all treatment-seeking populations or to non-treatment seeking individuals and this work highlights the need for longitudinal studies recruiting larger sample sizes in individuals with AUD of varying severity. A larger sample size will also help us evaluate the relationship between SRI and heavy drinking days. Another future direction is to evaluate whether treatments for sleep targeting an improvement in their SRI lead to a reduction in the number of heavy drinking days. These directions may help us understand SRI in those who do not seek treatment for their drinking or wish to drink in moderation, as they are more likely to seek treatment for their sleep problems rather than for their drinking.

## Conclusions

Given the advances of wearable devices in the past decade and the utility of being able to track a person’s sleep/wake routine, the SRI may be an important measure of sleep health to consider when trying to understand how sleep regularity might influence a person’s overall health, particularly in individuals with AUD who are working to maintain sobriety following inpatient treatment programs. These findings support the notion of SRI as a separate facet of sleep health worth investigating in at-risk, disease specific groups.

## Supplementary Information

Supplementary Information.

## Data Availability

The datasets used in this study are not publicly available due to ethical concerns regarding patient privacy and original patient consent. Data may be made available by requests directly to the corresponding authors.
