# Supplementary material for: A lower sleep regularity index (SRI) is associated with relapse in individuals with alcohol use disorder following inpatient treatment
Source: Sci Rep. 2022 Dec 14;12:21583. doi: 10.1038/s41598-022-26019-y (PMC9750995; doi:10.1038/s41598-022-26019-y)
Supplement: Supplementary file 1 — Supplementary Information. [file 41598_2022_26019_MOESM1_ESM.docx]

**Supplemental Table 1: Clinical and Sleep related measures**

| Assessment | Description | Timing of administration |
| --- | --- | --- |
| Pittsburgh Sleep Quality Index (PSQI) [24-26]  (Buysse et al., 1989; Backhuas et al., 2002; Doi et al., 2000) | The PSQI is a 19-item self-administered scale that assesses sleep quality and disturbance over a 30-day time interval and has been validated in normal populations as well as in populations with sleep disorders and psychiatric conditions. The scale generates a global score (0-21) and seven “component” scores (on a scale of 0-3 each); Subjective Sleep Quality, Sleep Latency, Sleep Duration, Habitual Sleep Efficiency, Sleep Disturbances, Use of Sleep Medication, and Daytime Drowsiness. A global score of 5 or higher is indicative of poor sleep quality with <5 being indicative of good sleep quality. Internal consistency and reliability coefficient range from 0.80 to 0.83 for its seven components 20-22 | Day 28 of inpatient stay or just prior to discharge. |
| Epworth Sleepiness Scale (ESS) [27, 28]  (Johns, 1991; Johns, 1992; Johns, 1993) | The ESS assesses excessive daytime sleepiness over the previous week. Individuals self-report their usual chances of dozing off or falling asleep in eight distinct activities common to everyday life. The ESS has an internal consistency and reliability coefficient between 0.70 and 0.88. A score higher than 10 indicates excessive day time sleepiness. | Within 7 days of discharge and 4-6 weeks post discharge. |
| Dysfunctional Beliefs and Attitudes about Sleep (brief version; DBAS-16) [29]  (Morin, Vallieres, & Ivers, 2007) | This 16-item brief version of the original 30-item scale assesses sleep-related cognitions including faulty beliefs and appraisals, unrealistic expectations, and perceptual and attention bias. Participants rate their agreement or disagreement on a 10-point Likert scale, where higher scores indicate stronger endorsement of dysfunctional beliefs and attitudes about sleep. The DBAS has an internal consistency of 0.77 for clinical and 0.79 for research samples. | Within 7 days of discharge and 4-6 weeks post discharge. |
| Self-Efficacy for Sleep Scale (SE-S) [30, 31]  (Rutledge, La Guardia, & Bluestein, 2013; Fichten, Libman, Creti, Amsel, Sabourin, Brender, & Bailes, 2001) | The SE-S measures individuals’ level of confidence in performing behaviors that might be helpful in initiating sleep. This nine-item scale has a score ranging from 9 to 45 with higher scores indicating greater confidence. The scale has established test-retest reliability as well as internal reliability ranging from 0.71 to 0.86. The concurrent validity of this scale has also been established by comparison with PSQI, sleep diaries, and objective sleep measures. | Within 7 days of discharge and 4-6 weeks post discharge. |
| Penn Alcohol Craving Sale (PACS) [19]  ( Flannery, Volpicelli, & Pettinati, 1999) | The PACS is a five-item scale measuring alcohol craving. The questions ask about frequency, intensity, and duration of thoughts about drinking, the ability to resist drinking, and average craving level over the past week. The PACS has established construct, predictive and discriminant validity as well as excellent internal consistency (Cronbach's alpha = 0.92)29 | Weekly throughout admission and 4-6 weeks post discharge. |
| Comprehensive Psychopathological Rating Scale (CPRS) [20-22]  (Åsberg, Montgomery, Perris, Schalling, & Sedvall, 1978; BSA; Tyrer, Owen, & Cicchetti, 1984; MADRS; Montgomery & Åsberg, 1979) | Assesses the severity of psychiatric symptoms and observed behaviors. Nineteen items correspond to two subscales of the CPRS: the Brief Scale for Anxiety (BSA) and the Montgomery Asberg Depression Rating Scale (MADRS).  The BSA is a 10-item measure that assesses pathological anxiety alone or in combination with other psychological or medical disorders 30  The MADRS is a 10-item measure that evaluates core symptoms of depression. Nine items of this scale are self-reported while one is based on the rater’s observation during the interview 31. | Weekly throughout admission. After discharge the BSA and MADRS were administered on weeks 1, 2, 4, 8 and 12 (as part of the aftercare program for the NIAAA screening protocol). Nurses on the unit collected data for BSA and MADRS from participants who came to the Clinical Center for their after care appointments on weeks 1, 2, 4, 8 and 12 post discharge. |
| Structured Clinical Interview for the Diagnostic and Statistical Manual of Mental Disorders (DSM-IV or DSM-5) (SCID) [23] | The SCID is an 11 module, widely used standard semi structured clinical interview to establish criteria for psychiatric diagnoses. Trained professionals or clinicians conduct interviews and final diagnoses are determined via a consensus process involving trained psychiatrists. For this analysis, diagnosis of one or more mood and/or anxiety disorders were used. | At the time of inpatient admission |

**Supplemental Table 2: Subjective sleep measures**

| ***Measure***  ***(mean* ± SD)** | ***All participants***  ***(n=77)*** | ***Non-relapse***  ***(n=59)*** | ***Relapse***  ***(n=18)*** | ****p-value*** |
| --- | --- | --- | --- | --- |
| **PSQI** | 7.28 ± 3.8 | 6.88 | 8.86 | .076 |
| **% Sleep efficiency** | 81.20 ±14.84 | 83.11 | 74.32 | **.036** |
| **Epsworth sleepiness** | 5.7 ± 3.7 | 5.94 | 5.26 | .532 |
| **Dysfunctional beliefs and attitudes sleep** | 3.88 ± 2.07 | 3.84 | 4.24 | .507 |
| **Self-efficacy for sleep** | 28.8 ±7.56 | 29.42 | 26.80 | .239 |
| **Hours in bed** | 8.2 ±1.6 | 8.15 | 8.42 | .567 |

*p-value reports the significance level of the difference of subjective sleep measure between individuals who relapsed and those who did not.

**Supplemental Figure 1: Total SRI compared to subjective sleep measures**


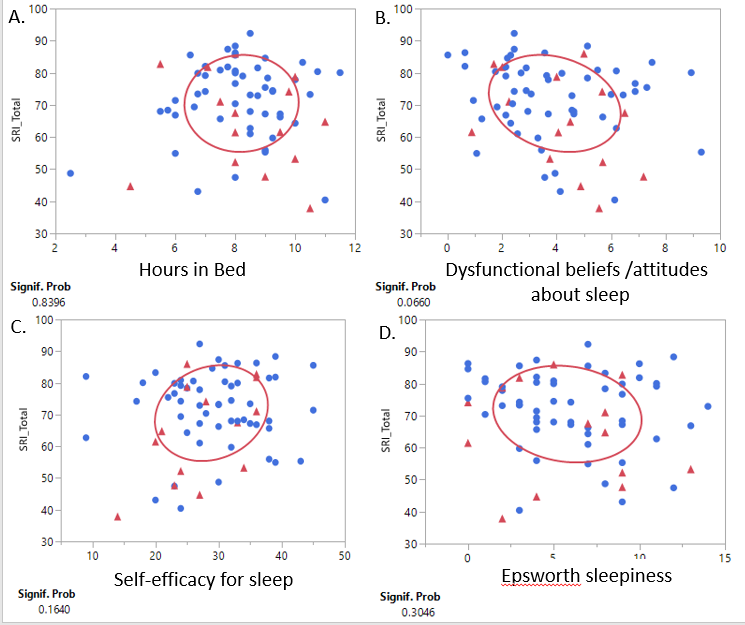


Total SRI (y-axis) versus A) Hours in bed (x-axis), B) dysfunctional beliefs about sleeping (DBAS) (x-axis), C) Self-efficacy for sleep (x-axis), D) Epsworth sleepiness scale (x-axis). No significant relationships observed.

**Supplemental Figure 2: Total SRI versus average nighttime light and average daytime light**


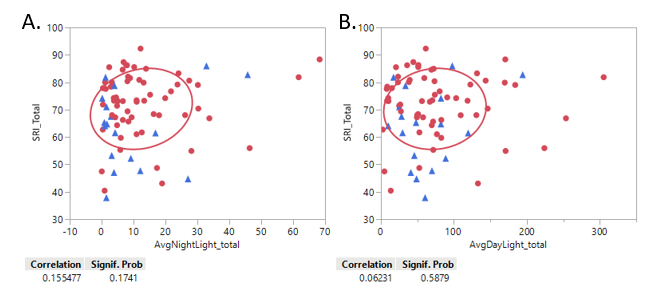


Total SRI versus average nightly light exposure. (A) Average nightly light exposure (x-axis) (B) average daily light exposure compared to total SRI (y-axis). Ellipse enclosed 50% of the data and shows correlation between the two measures. Red solid circles are individuals who did not relapse, and blue solid triangles are individuals who relapsed. No significant association observed between SRI and light exposure.

**Supplemental Figure 3: Alcohol consumption and days until relapse events in the group who experienced a relapse event (n=18)**


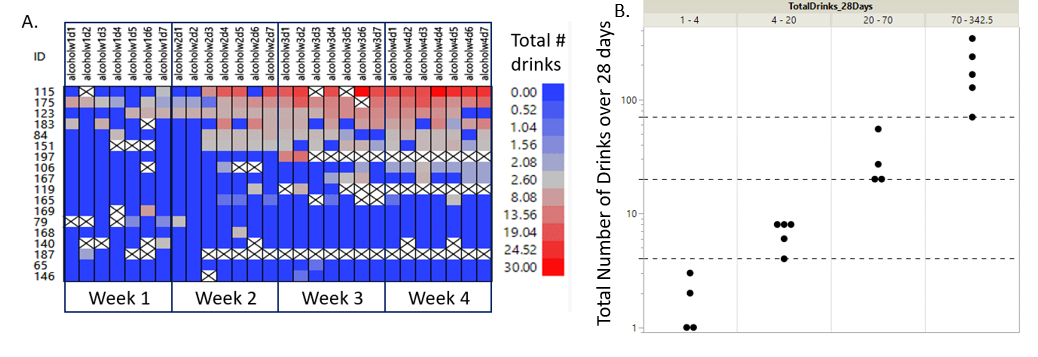


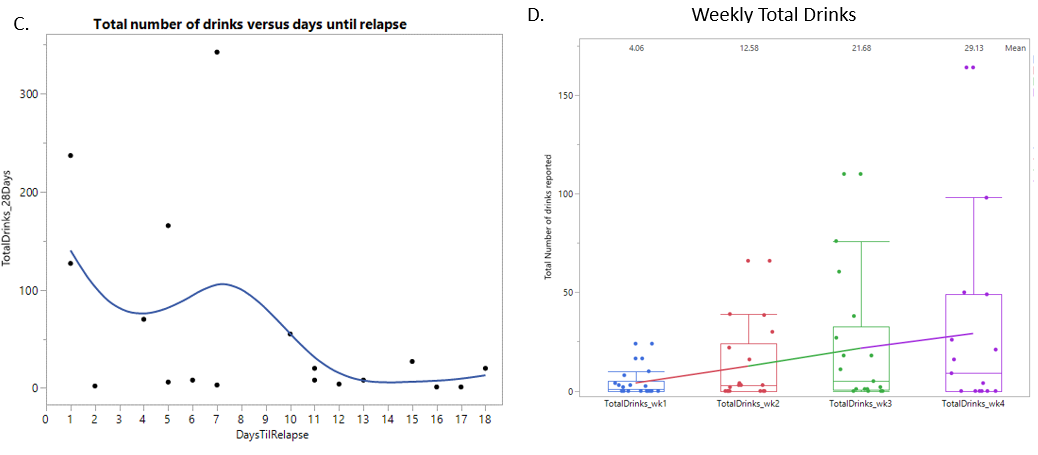


For individuals who had a relapse event, A) the total number of drinks consumed by each person for each day. The heat map shows the total drinks consumed over the period ranging from 0 to 30 for any given day. An X indicates no data provided for that day. X-axis above indicates week 1- day 1-7 through week 4- day 1-7. Y-axis indicates patient id of the relapse group ; B) total drinks across 28 days binned by four different groups of individuals who consumed anywhere from 1 to <4 drinks (n=4), ≥4 to <20 drinks (n=5), ), ≥20 to <70 drinks (n=4) and ≥70 to <342.5 drinks (n=5) over the 28 day period; C) Total number of drinks consumed versus the number of days until a relapse event occurred. Spline line fitted across the plot showing a linear downward trend; D) Total number of drinks per person at each week over the 28-day period. Colors indicate the total drinks at each week (i.e., blue is week 1, red is week 2, green is week 3 and purple is week 4).
